# A Double-Negative Feedback Interaction between miR-21 and PPAR-α in Clear Renal Cell Carcinoma

**DOI:** 10.3390/cancers14030795

**Published:** 2022-02-04

**Authors:** Marine Goujon, Justine Woszczyk, Kelly Gaudelot, Thomas Swierczewski, Sandy Fellah, Jean-Baptiste Gibier, Isabelle Van Seuningen, Romain Larrue, Christelle Cauffiez, Viviane Gnemmi, Sébastien Aubert, Nicolas Pottier, Michaël Perrais

**Affiliations:** 1 Univ. Lille, CNRS, Inserm, CHU Lille, UMR9020-U1277—CANTHER—Cancer Heterogeneity Plasticity and Resistance to Therapies, F-59000 Lille, France; marine.goujon@univ-lille.fr (M.G.); justine.woszczyk@gmail.com (J.W.); kelliii@hotmail.fr (K.G.); thomas.sw@hotmail.fr (T.S.); sandy.fellah@univ-lille.fr (S.F.); jeanbaptiste.gibier@chru-lille.fr (J.-B.G.); isabelle.vanseuningen@inserm.fr (I.V.S.); romain.larrue@univ-lille.fr (R.L.); christelle.cauffiez@univ-lille.fr (C.C.); viviane.gnemmi@chru-lille.fr (V.G.); sebastien.aubert@chru-lille.fr (S.A.); nicolas.pottier@univ-lille.fr (N.P.); 2 CHU Lille, Service d’Anatomo-Pathologie, F-59000 Lille, France; 3 CHU Lille, Service de Toxicologie et Génopathies, F-59000 Lille, France

**Keywords:** miR-21, PPAR-α, renal cancer, ccRCC, lipid, NF-κB, AP-1, transcriptional regulation

## Abstract

**Simple Summary:**

Clear cell renal cell carcinoma (ccRCC) is the main histotype of kidney cancer, which is typically highly resistant to conventional systemic therapies and also known for abnormal lipid accumulation. Identifying the actors and deciphering the molecular mechanisms that lead to tumor progression is an important step in the development of new therapeutic strategies to cure ccRCC. In this context, we focused our attention on miR-21, an oncogenic miRNA upregulated in many solid tumors, and peroxysome proliferator-activated receptor-α (PPAR- α), the master regulator of lipid metabolism and one target of miR-21. In this study, our data show a double-negative feedback interaction between PPAR-α and miR-21. Thus, miR-21 silencing could be therapeutically exploited to restore PPAR-α expression and consequently inhibit the oncogenic events mediated by the aberrant lipid metabolism of ccRCC.

**Abstract:**

Clear cell renal cell carcinoma (ccRCC) is the main histotype of kidney cancer, which is typically highly resistant to conventional therapies and known for abnormal lipid accumulation. In this context, we focused our attention on miR-21, an oncogenic miRNA overexpressed in ccRCC, and peroxysome proliferator-activated receptor-α (PPAR- α), one master regulator of lipid metabolism targeted by miR-21. First, in a cohort of 52 primary ccRCC samples, using RT-qPCR and immunohistochemistry, we showed that miR-21 overexpression was correlated with PPAR-α downregulation. Then, in ACHN and 786-O cells, using RT-qPCR, the luciferase reporter gene, chromatin immunoprecipitation, and Western blotting, we showed that PPAR-α overexpression (i) decreased miR-21 expression, AP-1 and NF-κB transcriptional activity, and the binding of AP-1 and NF-κB to the miR-21 promoter and (ii) increased PTEN and PDCD4 expressions. In contrast, using pre-miR-21 transfection, miR-21 overexpression decreased PPAR-α expression and transcriptional activity mediated by PPAR-α, whereas the anti-miR-21 (LNA-21) strategy increased PPAR-α expression, but also the expression of its targets involved in fatty acid oxidation. In this study, we showed a double-negative feedback interaction between miR-21 and PPAR-α. In ccRCC, miR-21 silencing could be therapeutically exploited to restore PPAR-α expression and consequently inhibit the oncogenic events mediated by the aberrant lipid metabolism of ccRCC.

## 1. Introduction

An aberrant metabolism is a hallmark of tumors [1], and alterations in lipid metabolism represent one of the most important features of renal cell carcinoma (RCC), especially the clear cell type (ccRCC), which accounts for 70–85% of all RCC cases [2]. Renal neoplastic cells harness lipid metabolism to sustain uncontrolled proliferation, avoid cell death, and seed in secondary organs. Although ccRCC is known for abnormal lipid accumulation of cholesterol, cholesterol esters, and neutral lipids (triglycerides) [3], the underlying molecular mechanisms remain unclear. Furthermore, most patients with ccRCCs harbor chromosomal 3p loss and genomic mutations in the Von Hippel–Lindau Tumor Suppressor (VHL) allele, followed by the secondary loss of multiple tumor suppressor genes including PBRM1, SETD2, PTEN, and/or TP53 [4,5,6,7]. ccRCCs are also known to be highly resistant to conventional cytotoxic, radiation, and hormone therapies, and nephrectomy is the only therapeutic option used to cure early and local ccRCCs. At diagnosis, 25% of patients present a metastatic disease, and one third develop metastasis after surgery. The current treatment options offered to metastatic patients include inhibitors of (i) tyrosine kinases, (ii) mTORC signaling, and (iii) immune control checkpoints. Despite the initial response, most metastatic ccRCC patients will develop resistance to these targeted therapies [4,5,6,7]. Therefore, deciphering the molecular mechanisms underlying renal tumor progression is urgently needed to develop new therapeutic strategies to cure ccRCC.

MicroRNAs (miRNAs) are a class of small non-coding RNAs of 19–22 nucleotides in length that repress translation and/or induce degradation of target genes, typically by binding to their 3′-UTR. miRNAs are major regulators of gene expression and are involved in a variety of biological processes such as differentiation, proliferation, metabolism, and apoptosis [8,9]. Furthermore, the aberrant expression of miRNAs has been shown to play a causative role in many human diseases, and specific miRNA expression patterns have been associated with the initiation and progression of various complex diseases including cancer.

miR-21, one of the earliest miRNAs identified as an “OncomiR” [10], is the most commonly upregulated miRNA in both solid and hematological malignancies [11]. In our previous works, we provided evidence that miR-21 represents an attractive therapeutic target in renal cancer given its multifaceted and central role in the molecular and cellular events associated with renal cancer progression and drug resistance [12]. Nevertheless, whether miR-21 also contributes to the dysregulation of lipid metabolism in cancer cells is currently poorly understood.

Peroxysome proliferator-activated receptor-α (PPAR- α), the master regulator of lipid metabolism, is a nuclear receptor that acts as a ligand-activated transcription factor. PPAR-α activates numerous enzymatic pathways involved in fatty acid (FA) uptake, intracellular transport, FA activation and β-oxidation, and lipoprotein/cholesterol metabolism [13,14]. PPAR-α regulates the expression level of its targets in a transcriptional manner through heterodimerization with the retinoid X receptor (RXR). Once activated by a ligand, this complex binds to the peroxisome proliferator response element (PPRE) located in the promoter region of the target genes and modulates their transcription. PPAR-α can also negatively regulate transcription mediated by AP-1 and NF-κB by protein–protein interactions. Interestingly, PPAR-α is an established target of miR-21 [15,16,17], and AP-1 and NF-κB have been shown to be involved in the transcriptional control of miR-21 [18,19], thus suggesting a negative regulatory loop between miR-21 and PPAR-α. Herein, we provide evidence for this regulatory mechanism by showing (i) the concomitant altered expression of both PPAR-α and miR-21 in a cohort of 52 primary ccRCCs, (ii)the transcriptional regulation of miR-21 by PPAR-α through decreased binding of AP-1 and NF-κB to the miR-21 promoter, and (iii) the increased transcriptional activity and expression of PPAR-α and key enzymes involved in FA oxidation (FAO) following miR-21 silencing.

## 2. Material and Methods

### 2.1. Clinical Specimen

A total of 52 primary ccRCC samples with healthy tissues were collected after surgery and stored in the Tumor Bank and Tissue Collection of the Department of Pathology of Lille Hospital. A written consent form was obtained from each patient, and the study was approved by the scientific committee of the institute Tumorothèque du CHRU de Lille (approval number: CSTMT078). The cohort has been previously described in [12]. All the procedures performed in the studies involving human participants were carried out in accordance with the ethical standards of the institutional research committee and the Declaration of Helsinski 1964 and its later amendments or comparable ethical standards.

### 2.2. Immunohistochemistry

The immunochemistry protocol for PPAR-α (clone ab8934, abcam, Paris, France; 1/200) was performed as previously described [20,21]. An IgG control antibody was used for immunohistochemical analysis and did not show specific staining.

### 2.3. Cell Lines and Culture Conditions

The HK-2 human proximal tubule epithelial cell line, ACHN, and 786-O human renal cancer cell lines were purchased from the American Type Culture Collection (ATCC, Manassas, VA, USA). The RCC10 and RCC4 human renal cancer cell lines were a gift from Dr. D. Bernard (Inserm U1052—CNRS UMR5286, Centre de Recherche en Cancérologie de Lyon, Lyon, France) [22]. The ACHN, 786-O, and RCC10/RCC4 cells were cultured, respectively, with MEM, DMEM, and RPMI-1640 medium, supplemented with 10% heat-inactivated FBS, 1% l-glutamine, and 1% penicillin/streptomycin, while the HK2 cells were cultured with Keratinocyte Serum-Free Medium supplied with 0.05 mg/mL Bovine Pituitary Extract, 5 ng/mL human recombinant epidermal growth factor, and 1% penicillin/streptomycin. The cells were treated with 400 nM or 1 mM of GW7647, a highly selective agonist of PPAR-α (Merck, Darmstadt, Germany).

### 2.4. Quantitative RT-PCR

Total RNA was extracted from the tissue and cell samples with the RecoverAll Total Nucleic Acid isolation kit (Ambion, Thermo Fisher Scientific, Illkirch-Graffenstaden, France) and the miRNeasy Mini kit (Qiagen, Courtaboeuf, France), respectively.

#### 2.4.1. Mature miRNA Expression 

Retrotranscription was performed on 5 ng of total RNA with a TaqMan probe (hsa-miR-21: 000397; RNU6B: 001093; RNU48: 001006 (Thermo Fisher Scientific, Applied Biosystem, Illkirch-Graffenstaden, France) according to TaqMan microRNA Reverse Transcription Kit protocol (Applied Biosystem). The qPCR reaction was performed using the TaqMan Gene Expression Master Mix (Applied Biosystem) following the manufacturer’s protocol using the CFX96 Real-Time PCR system (Bio-Rad, Marnes La Coquette, France). The miRNA expression data were normalized to the expression of RNU6B for the tissue samples and to the expression of RNU48 for the cell samples. The expression levels of mature miR-21 were calculated based on the comparative threshold cycle method (2^−^^ΔΔ^^CT^) [23].

#### 2.4.2. Gene Expression 

Retrotranscription was performed on 1 μg of total RNA with the High Capacity cDNA Reverse Transcription Kit (Thermo Fisher Scientific, Applied Biosystem, Illkirch-Graffenstaden, France). The expression levels of PPAR-α, ACOX1, CPT1, and SLC22A5 were analyzed using the TaqMan Expression Assay (Thermo Fisher Scientific, Illkirch-Graffenstaden, France). The qPCR reaction was performed using the TaqMan Gene Expression Master Mix (Applied Biosystem) following the manufacturer’s protocol using the CFX96 Real-Time PCR system (Bio-Rad). The mRNA expression data were normalized to the expression of PPIA (cyclophilin A). The expression levels of mature miR-21 were calculated based on the comparative threshold cycle method (2^−^^ΔΔ^^CT^) [23].

### 2.5. Western Blotting

Total cellular extracts and Western blotting were performed as previously described [24] using specific primary antibodies: PPAR-α (Cayman Chemical, Ann Arbor, MI, USA), PTEN (Cell Signaling), PDCD4 (Cell Signaling, Danvers, MA, USA); β-Actin (Sigma-Aldrich, Saint-Quentin-Fallavier, France). Peroxydase-conjugated secondary antibodies (Sigma-Aldrich, Saint-Quentin-Fallavier, France) were used, and immunoreactive bands were visualized using the West Pico chemoluminescent substrate (Thermo Scientific, Pierce, Illkirch-Graffenstaden, France). Chemo-luminescence was visualized using an LAS4000 apparatus (Fujifilm, Courbevoie, France). The original Western Blotting data is shown in Supplementary Materials (Figure S1).

### 2.6. Transient Transfections

Transient transfections and co-transfections were performed with Lipofectamine LTX (Invitrogen) as previously described [16]. The plasmids used in this study were: pSG5-EV (empty vector), pSG5-PPAR-α (a gift from Professor B. Staels, Inserm U1011, CHU de Lille, Institut Pasteur de Lille, Lille, France), pGL3-miR-21-Luc (−1656/+24) miR-21 promoter, [18], κB-Luc, AP1-Luc, and J6-PPRE-TK-Luc (a gift from Professor B. Staels). To determine the promoter activity, 24 or 48 h after transfection, the cell lysates were determined for firefly and *Renilla* luciferase activities with the Secrete-Pair^TM^ Dual Luminescence Assay Kit (Promega, Charbonnières-Les-Bains, France). The results were expressed as relative luciferase units normalized to *Renilla* luciferase [25].

### 2.7. Chromatin Immunoprecipitation

The 786-O and ACHN cells (1.0 × 10^6^) were fixed for 10 min at room temperature in 1% (*v*/*v*) formaldehyde and processed for ChIP analysis as previously described [26]. The specific antibodies used were: anti-c-jun (D) and anti-NF-κB p65 (H-286) (Santa Cruz Biotechnology, Heidelberg, Germany). qPCR was performed using the SsoFast EvaGreen supermix (Bio-Rad) and the following primers: AP-1 Forward: 5′-TAAGGATGACGCACAGATTGTC-3′; AP-1 Reverse: 5′-TCAGAAGTCCCACATTTATCACC-3′; NF-κB Forward: 5′-GGAGTGGATGGGTTCTGCCTTA-3′; and NF-κB Reverse: 5′-CAAGGTGGATTGCATCGAGG-3′.

### 2.8. Modulating miR-21 Expression in Renal Cancer Cells

In order to upregulate miR-21 expression and downregulate miR-21 expression in renal cancer cells, premiR hsa-miR-21-5p (Ambion, Thermo Fisher Scientific, Illkirch-Graffenstaden, France) and miRCURY LNA Inhibitor hsa-miR-21-5p (Exiqon, Vedbaek, Denmark) were transfected, respectively. The control cells were transfected with premiR miRNA Precursor Molecules-Negative Control #2 (Ambion) or with miRCURY LNA Power Inhibitor Control (Exiqon) [12]. Reverse transfection was performed with Lipofectamine RNAiMAX Reagent (Invitrogen, Thermo Fisher Scientific, Illkirch-Graffenstaden, France) according to the manufacturer’s instruction.

### 2.9. Statistical Analysis

The data are presented as means ± SEM (standard error mean). The statistical analyses were performed with GraphPad Prism software (GraphPad Software, San Diego, CA, USA). Differences between multiple groups were assessed by one-way analysis of variance (ANOVA) followed by Bonferroni’s multiple comparison test. Statistical differences between each two groups were determined by the Student’s *t*-test. Differences were considered significant when *p* < 0.05.

## 3. Results

### 3.1. miR-21 Overexpression Is Correlated with the Downregulation of PPAR-α Expression in Clear Renal Cell Carcinoma

**Figure 1:**
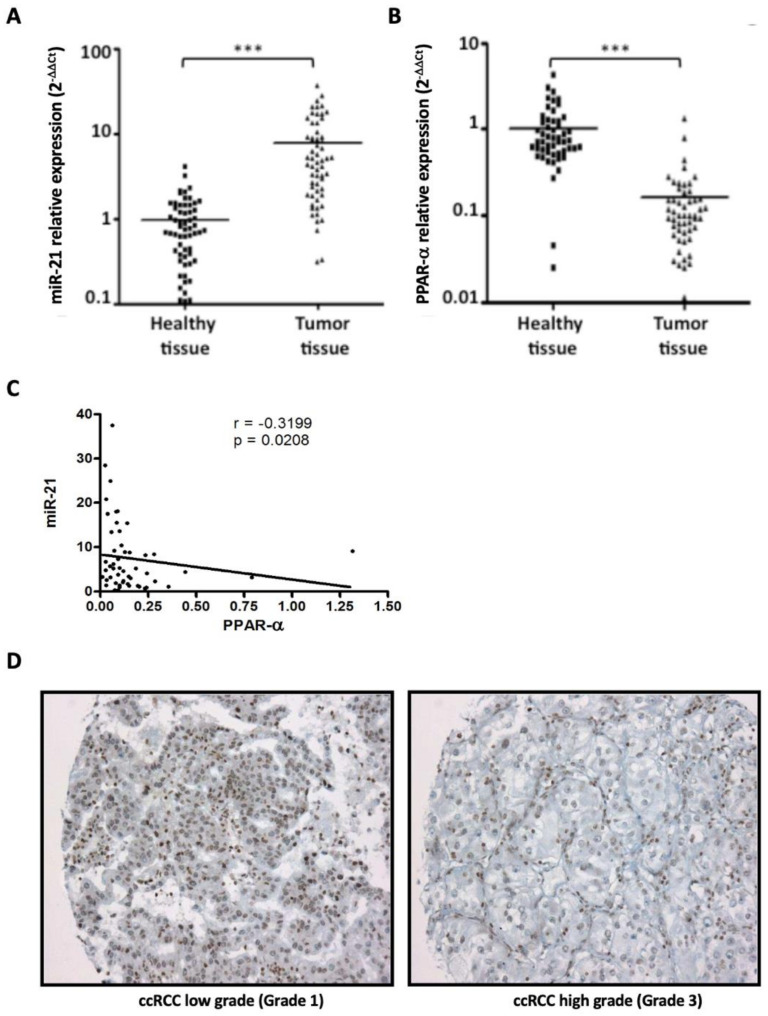
miR-21 is significantly upregulated and PPAR-*α* downregulated in ccRCC human tissues compared to the paired healthy tissues. (**A**) miR-21 and (**B**) PPAR-*α* expressions in the tumor and adjacent healthy renal tissues of a cohort of 52 cRCC patients were determined by qRT-PCR. RNU6B and PPIA were used as internal controls, respectively. The average expression of miR-21 and PPAR-*α* in ccRCC is shown in relation to the value obtained for normal kidney tissue (*** *p* < 0.001). (**C**) The correlation between miR-21 and PPAR-*α* expressions in ccRCC was analyzed with Spearman’s test. (**D**) The immunohistochemistry study preformed using an anti-PPAR-*α* antibody (magnification: ×200).

First, using RT-qPCR, we examined miR-21 and PPAR-α expression in 52 paired ccRCC tumor tissues and matched adjacent non-tumor tissues. miR-21 expression was significantly upregulated in ccRCC tissues compared to the normal renal tissues (14.48 ± 2.6 vs. 0.97 ± 0.1, respectively; *p* < 0.001; Figure 1A), whereas PPAR-α expression was significantly decreased (0.16 ± 0.03 vs. 1.03 ± 0.12, respectively; *p* < 0.001; Figure 1B). Then, we showed that miR-21 overexpression was correlated with PPAR-α downregulation in ccRCC (Spearman’s test: r = −0.32; *p* = 0.0208; Figure 1C). The immunohistochemistry analyses showed very low expression of PPAR-α in neoplastic cells except in a few ccRCC low-grade tumors (nucleolar grade 1–2 as described by the World Health Organization/International Society of Urological Pathology (WHO/ISUP)) (Figure 1D). Furthermore, in a few ccRCC high-grade tumors (nucleolar grade 3–4), a nuclear expression of PPAR-α was observed, mainly restricted to tumor-infiltrated inflammatory cells, in particular lymphocytes (Figure 1D). Overall, cancer cells in ccRCC exhibited high levels of miR-21, and the expression of PPAR-α was nearly undetectable. 

### 3.2. miR-21 and PPAR-α Expressions in Renal Cellular Models

**Figure 2:**
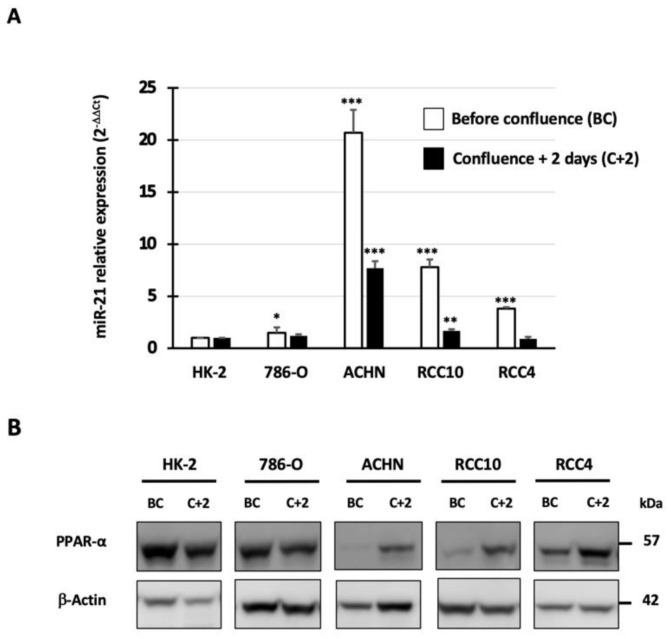
miR-21 and PPAR-α expressions in HK-2 and renal cancer cell lines before confluence (BC) and two days after confluence (C + 2). (**A**) Using RT-qPCR, miR-21 expression was determined in the 786-O, ACHN, RCC10, and RCC4 renal cancer cells as compared to normal HK-2 renal epithelial cells. RNU48 was used as an internal control. The values are means ± SEM and represent at least three separate experiments (* *p* < 0.05, ** *p* < 0.01, and *** *p* < 0.001). (**B**) Western blotting was performed on whole cell extracts. Antibodies against PPAR-α and β-actin were used.

To further investigate the relationship between miR-21 and PPAR-α, we first assessed the expression level of miR-21 and PPAR-α in human proximal tubule epithelial cell line HK-2 and in four human ccRCC cell lines. Using RT-qPCR, we showed that miR-21 expression was significantly upregulated before confluence in the 786-O, ACHN, RCC10, and RCC4 cancer cell lines compared to HK-2 cells (*p* < 0.05 and *p* < 0.001; Figure 2A). The ACHN cells expressed high level of miR-21 (21-fold, *p* < 0.001), whereas 786-O expressed lower levels (1.7-fold, *p* < 0.05) compared to HK-2 cells. Two days after the cells reached confluence, miR-21 expression decreased in all the cancer cell lines; however, the Western blot analysis revealed induction of PPAR-α (Figure 2B). Therefore, variation in miR-21 expression directly impacts PPAR-α levels only in ccRCC cell models. 

### 3.3. PPAR-α Expression and/or Activation Decreases the Expression of miR-21

**Figure 3:**
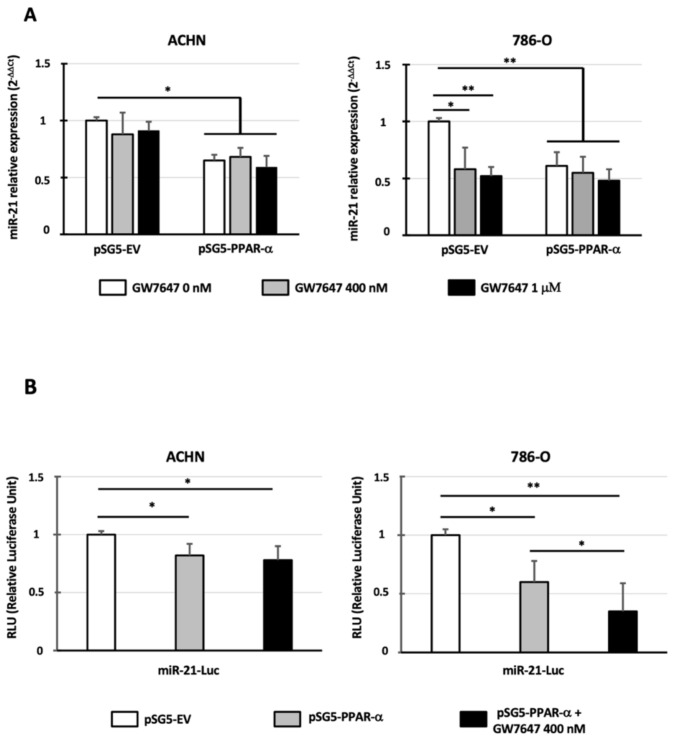
PPAR-α expression and/or activation decreases miR-21 expression and transcription. (**A**) ACHN and 786-O cells were transfected with pSG5-EV (empty vector) or pSG5-PPAR-α expression vectors. At 24 h after transfection, the cells were incubated with 0, 400 nM, or 1 μM of GW7647. At 48 h after transfection, miR-21 expression was determined by qPCR. RNU48 was used as an internal control. (**B**) ACHN and 786-O cells were co-transfected with miR-21-Luc promoter and pSG5-EV (empty vector) or pSG5-PPAR-α expression vectors. At 24 h after transfection, the cells were incubated with 0 or 400 nM of GW7647. At 48 h after transfection, luciferase activity was determined. The values obtained with the empty vector were referred to as 1. The values are means ± SEM and represent at least four separate experiments (* *p* < 0.05 and ** *p* < 0.01).

To investigate whether the modulation of PPAR-α activity can modulate the expression of miR-21, ccRCC cell models were exposed to GW7647, a highly selective PPAR-α agonist, and also transiently transfected by the pSG5-PPAR-α expression vector containing PPAR-α cDNA without a putative miR-21 binding site in its 3′-UTR. In ACHN cells that expressed a high level of miR-21, PPAR-α activation by GW7647 had no effect on miR-21 expression, whereas PPAR-α overexpression significantly decreased miR-21 levels (32–41% inhibition, *p* < 0.05; Figure 3A). In 786-O cells that expressed a very low level of miR-21, GW7647 alone was sufficient to significantly decrease miR-21 expression (42–48% inhibition, *p* < 0.05 and *p* < 0.01; Figure 3A). Similarly, the forced expression of PPAR-α also decreased miR-21 expression by 39% (*p* < 0.01), but no additive effect was observed when the cells were treated with the PPAR-α agonist. In conclusion, variation in PPAR-α expression and/or activity is able to decrease miR-21 expression in renal cancer cells.

### 3.4. PPAR-α Expression and/or Activation Decreases the Expression of miR-21 at the Transcriptional Level

To further define how PPAR-α downregulates miR-21, we cloned the −1656/+24 miR-21 promoter described by Fujita et al. [18] in a pGL3 basic luciferase reporter vector. The co-transfection experiments performed in the ACHN and 786-O cells showed that PPAR-α overexpression decreased miR-21 promoter transcriptional activity (18% and 40% inhibition, respectively; Figure 3B). This inhibition increased significantly after the GW7647 treatment only in the 786-O cells (65% inhibition). Thus, PPAR-α overexpression decreases miR-21 promoter transcriptional activity in renal cancer cells. 

### 3.5. PPAR-α Expression and/or Activation Decreases AP-1 and NF-κB Transcriptional Activity and Binding to miR-21 Promoter

**Figure 4:**
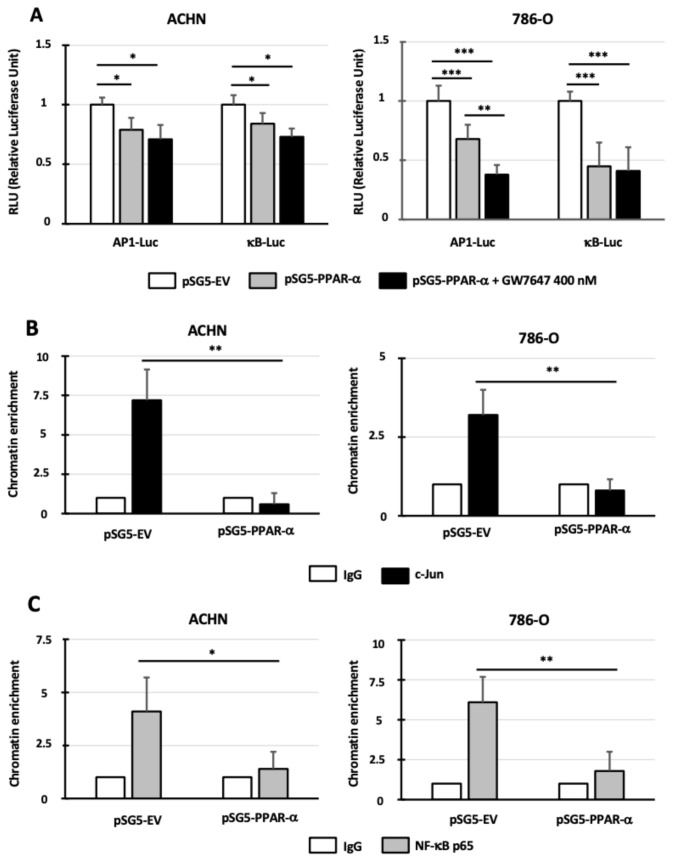
PPAR-α expression and/or activation decreases AP-1 and NF-κB transcriptional activity and binding to miR-21 promoter. (**A**) ACHN and 786-O cells were co-transfected with pSG5-EV (empty vector) or pSG5-PPAR-α expression vectors and κB-Luc or AP1-Luc synthetic promoters. At 24 h after transfection, the cells were incubated with 0 or 400 nM of GW7647. At 48 h after transfection, luciferase activity was measured. Luciferase activity in cells transfected with pSG5-EV was set as 1. (**B**,**C**) Soluble chromatin from the ACHN and 786-O cells transfected with pSG5-EV or pSG5-PPAR-α expression vectors was immunoprecipitated with control immunoglobulin G (IgG) and anti-c-jun (**B**) or anti-NF-κB p65 antibodies (**C**). The precipitated DNA samples were amplified by PCR with pairs of primers flanking the binding site regions for AP-1 and NF-κB. The results were expressed as the percentage of input. The values obtained with the empty vector were referred to as 1. The values are means ± SEM and represent at least three separate experiments (* *p* < 0.05, ** *p* < 0.01, and *** *p* < 0.001).

Then, we focused our attention on the AP-1 and NF-κB transcription factors that (i) regulate miR-21 transcriptional activity [18,19], (ii) interact with those whose transcriptional activity is inhibited by PPAR-α via a transrepressive mechanism [8], and (iii) are overexpressed and activated in renal carcinogenesis [27,28]. To determine AP-1 and NF-κB activities in our cellular models, we investigated AP-1- and NF-κB-dependent transcription directly using AP1-Luc and κB-Luc reporter assays. In the ACHN cells, PPAR-α overexpression significantly decreased transcriptional activity mediated by AP-1 and NF-κB (≈20% inhibition, *p* < 0.05; Figure 4A), but GW7647 had no effect. In the 786-O cells, PPAR-α overexpression significantly decreased transcriptional activity mediated by AP-1 and NF-κB (32 and 55% inhibition, respectively, *p* < 0.001; Figure 4A). The treatment with the PPAR-α agonist increased transcriptional activity mediated by AP-1 (62% inhibition, *p* < 0.001). Then, we determined whether PPAR-α overexpression influenced the binding of AP-1 and NF-κB on the *miR-21* promoter. In the control conditions (pSG5-EV), the ChIP assays showed that, in the ACHN and 786-O cells, AP-1 interacted with the miR-21 promoter when we compared the chromatin enrichment in anti-c-jun and IgG conditions (7.2 ± 1.9 and 3.2 ± 0.8, respectively; *p* < 0.01). These interactions ceased when PPAR-α was overexpressed (pSG5-PPAR-α). The same results were obtained with the NF-κB transcription factor: in the ACHN and 786-O cells transfected with pSG5-EV, chromatin enrichment was observed in anti-NF-κB p65 vs. IgG conditions (4.1 ± 1.6 (*p* < 0.05) and 6.09 ± 1.5 (*p* < 0.01), respectively), whereas no significant enrichment was observed when the cells were transfected with the pSG5-PPAR-α expression vector. In conclusion, PPAR-α expression decreases AP-1 and NF-κB transcriptional activity and binding to miR-21 promoter.

### 3.6. PPAR-α Expression and/or Activation Increases the Expression of Two miR-21 Targets: PDCD4 and PTEN

**Figure 5:**
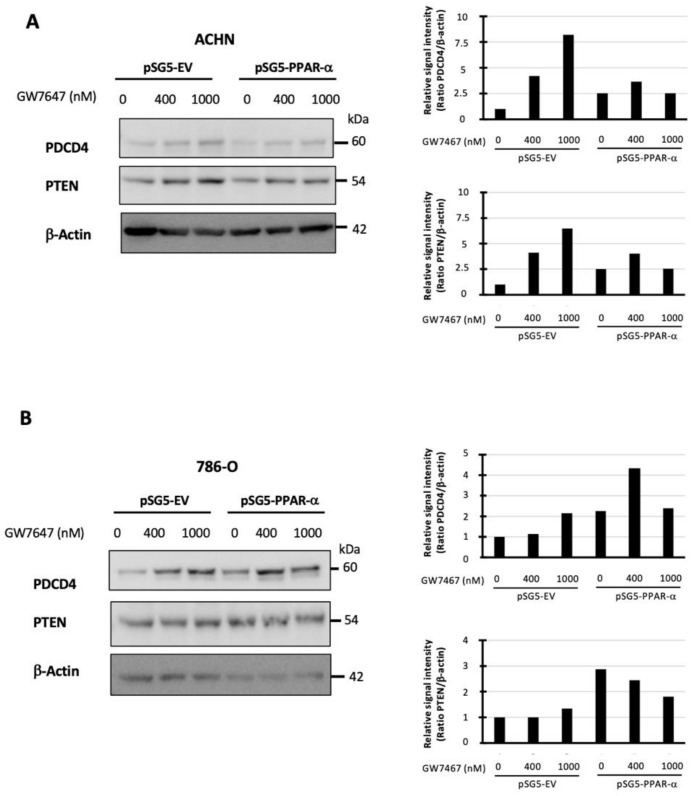
PPAR-α expression and/or activation increases the expression of two miR-21 targets: PDCD4 and PTEN in ACHN (**A**) and 786-O cells (**B**). Western blot performed on cell extracts obtained from ACHN and 786-O transfected with pSG5-EV (empty vector) or pSG5-PPAR-α expression vectors and treated for 24h after transfection with 0, 400 nM, or 1 μM of GW7647. The intensities of the signal were determined by densitometric scanning, expressed as the relative signal PDCP4/Actin and PTEN/Actin ratios and represented as histograms. Expression in the “pSG5-EV with no treatment” condition was arbitrarily set to 1.

After showing that PPAR-α decreased miR-21 expression directly at the transcriptional level, we evaluated the impact of PPAR-α overexpression and activation on the expression levels of two known miR-21 targets: PDCD4 and PTEN. In the ACHN and 786-O cells, PPAR-α overexpression increased PDCD4 (2.5-fold (Figure 5A) and 2.2-fold (Figure 5B)) and PTEN (2.5-fold (Figure 5A) and 2.9-fold (Figure 5B)) expression levels. Similar results were obtained when the cells were exposed to GW7647. Altogether, these results suggest that PPAR-α positively regulates PDCD4 and PTEN expressions.

### 3.7. Inhibition of miR-21 Expression Increases PPAR-α and PPAR-α Target Gene Expression

**Figure 6:**
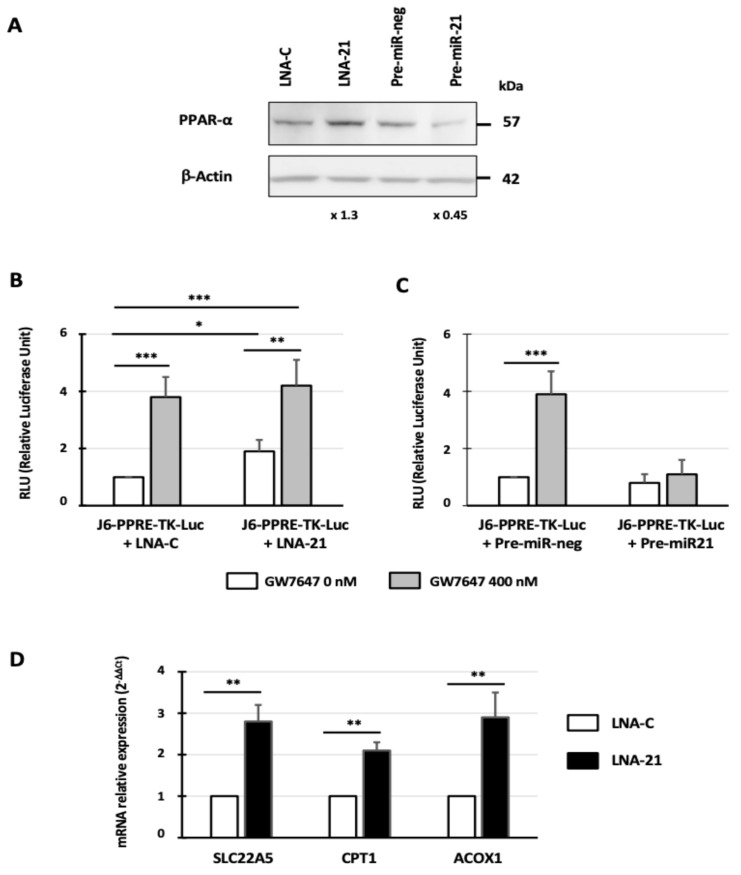
Inhibition of miR-21 expression increases PPAR-α and PPAR-α target gene expressions in 786-O cells. (**A**) Western blot performed on cell extracts obtained from 786-O cells transfected for 24h with 10 nM of LNA-C, LNA-anti-miR-21 (LNA-21), pre-miR-neg, or pre-miR-21. (**B**) The 786-O cells were co-transfected with J6-PPRE-TK-Luc reporter plasmid and 10 nM of LNA-C or LNA-anti-miR-21 (LNA-21). At 48 h after transfection, luciferase activity was measured. Luciferase activity in the cells co-transfected with J6-PPRE-TK-Luc and LNA-C was set as 1. (**C**) The 786-O cells were co-transfected with J6-PPRE-TK-Luc reporter plasmid and 10 nM of pre-miR-neg or pre-miR-21. At 48 h after transfection, luciferase activity was measured. Luciferase activity in the cells co-transfected with J6-PPRE-TK-Luc and pre-miR-neg was set as 1. (**D**) The 786-O cells were transfected with 10 nM of LNA-C or LNA-anti-miR-21 (LNA-21). At 48 h after transfection, SLC22A5, CPT1, and ACOX1 expressions were determined by RT-qPCR. PPIA was used as an internal control. The values are means ± SEM and represent at least three separate experiments (* *p* < 0.05, ** *p* < 0.01, and *** *p* < 0.001).

To further define how miR-21 downregulates PPAR-α, anti-miR-21 (LNA-21) or pre-miR-21 was transfected in the 786-O cells to either inhibit or overexpress miR-21, respectively [7]. Using RT-qPCR, PPAR-α expression was not affected when miR-21 was overexpressed or downregulated (data not shown). However, using Western blotting (Figure 6A), we showed that miR-21 downregulation using an LNA-21 strategy increases PPAR-α expression (1.3-fold), whereas miR-21 overexpression by transfection of pre-miR-21 decreases its expression (55% inhibition). Then, we evaluated the impact of miR-21 expression on PPAR-α signaling. First, we used the J6-PPRE-TK-Luc reporter plasmid in which the luciferase gene was under the control of the thymidine kinase promoter containing three PPRE elements. When the J6-PPRE-TK-Luc construct was co-transfected with LNA-21, luciferase activity was increased by 1.9-fold (*p* < 0.05) compared to LNA-C (Figure 6B). In both co-transfection conditions, the treatment with GW7647 significantly increased luciferase activities (3.8-fold and 4.2-fold, respectively; *p* < 0,001). By contrast, when the cells were co-transfected with J6-PPRE-TK-Luc and pre-miR-21, GW7647 had no effect on transcriptional activity mediated by PPAR-α. Then, to confirm that miR-21 plays a role in transcriptional activity mediated by PPAR-α, we evaluated the expression of three PPAR-α target genes involved in lipid metabolism: SLC22A5, CPT1, and ACOX1. When miR-21 was inhibited by LNA-21, their expression levels significantly increased (2.8-, 2.1-, and 2.9-fold, respectively; *p* < 0.01) (Figure 6D). In conclusion, miR-21 overexpression decreases PPAR-α expression and transcriptional activity mediated by PPAR-α. 

## 4. Discussion

Studies over the last decade have led to the concept that ccRCC, the most common and deadly type of cancer affecting the kidney, is a metabolic disease histologically characterized by large intracellular lipid deposits [3]. As ccRCC is typically highly resistant to conventional systemic therapies [2,5,6,7], the identification of new molecular mechanisms driving tumor progression is essential for the rational design of new therapeutic strategies to cure ccRCC. In this context, we focused our attention on miR-21, an established oncogenic miRNA commonly upregulated in many solid tumors such as breast, lung, and stomach cancers and hematological malignancies [10,11]. Mechanistically, miR-21 has been shown to influence cancer by targeting essential tumor-suppressive genes such as PTEN, BCL2, or PDCD4, thereby promoting tumor development by regulating many distinct carcinogenic pathways including those related to proliferation, apoptosis, angiogenesis, and metastasis [29]. For example, we previously showed that miR-21 is not only upregulated in ccRCC but is also involved in cancer progression (proliferation, migration, and invasion) and resistance to chemotherapy by controlling the expression of genes associated with multi-drug resistance and the apoptotic pathway [12]. Nevertheless, whether miR-21 also participates in the metabolic alterations characterizing ccRCC remains incompletely understood and is of particular interest, especially as this miRNA has been previously shown to target PPAR-α, the master regulator of lipid metabolism [15,16,17]. Furthermore, during kidney fibrosis, miR-21 overexpression contributes to fibrosis and epithelial injury by downregulating PPAR-α expression and increasing lipid accumulation [15,30,31,32]. In this study, based on our cohort of 52 paired ccRCC tumor tissues and matched adjacent non-tumor tissues, we showed the overexpression of miR-21 in ccRCC as well as an inverse correlation between miR-21 and PPAR-α expression. Consistent with Kim et al. [33], we also showed that the PPAR-α protein level was barely detectable in most ccRCC clinical samples, suggesting that miR-21 may influence PPAR-α expression and consequently its activity. As PPAR-α is a bona fide target of miR-21, we performed a series of experiments aiming to better characterize the regulatory mechanism shared by miR-21, PPAR-α, and lipid metabolism in ccRCC. 

PPAR-α, the master regulator of lipid metabolism, typically modulates gene expression by binding to specific DNA response elements located in the promoter region of the target genes as heterodimers with retinoid X receptors (RXRs) [13]. This usually enables PPAR-α to positively regulate gene networks involved in the control of lipid metabolism, in particular the transcriptional control of genes involved in peroxymal and mitochondrial FAO, fatty acid uptake, and triglyceride catabolism. PPAR-α is also directly involved in the negative regulation of proinflammatory genes in a ligand-dependent manner by antagonizing the activities of several transcription factors such as NF-κB and AP-1 by a less understood mechanism termed transrepression [13,34]. Here, we showed that PPAR-α negatively regulates miR-21 expression by a mechanism of transrepression involving AP-1 and NF-κB. Indeed, our results demonstrate that PPAR-α represses miR-21 expression by decreasing AP-1 and NF-κB transcriptional activity on the miR-21 promoter. This is consistent with previous studies that showed the overexpression and increased activity of AP-1 and NF-κB in ccRCC [27,28], as well as their ability to increase miR-21 transcription [18,19].

Finally, we showed that silencing miR-21 is sufficient to increase PPAR-α expression and activity. To further confirm the impact of miR-21 silencing on the metabolic changes induced by PPAR-α activation, we also assessed the expression level of three transcriptional targets of PPAR-α involved in lipid metabolism: SLC22A5, the carnitine transporter; CPT1, carnitine palmitoyltransferase 1, which transports FA to the mitochondria and ACOX1; and acyl-CoA oxidase 1, which is the first and rate-limiting enzyme in FAO [13,14]. Of particular interest, the inhibition of miR-21 expression significantly increased the expression of these genes, suggesting that miR-21 could contribute to lipid accumulation characterizing ccRCC. A study on more than 400 renal tumor samples showed the aberrant activation of PI3K/AKT and the expression of two top proteins correlated with worse survival: reduced AMP-activated kinase (AMPK) and increased acetyl-CoA carboxylase (ACC). Together, the downregulation of AMPK and the upregulation of ACC activity contribute to a metabolic shift towards increased FA synthesis [35]. Thus, ccRCC is characterized by a lipid storage phenotype. CPT1 is repressed by the hypoxia-inducible factor, the main signaling pathway involved in renal carcinogenesis, and reducing FAO. A lower expression of CPT1 is associated with poor patient outcomes [36]. Altering lipid metabolism may be a new therapeutic avenue for ccRCC [37,38,39]. In line with these studies, the miR-21-silencing strategy is of potential interest in ccRCC treatment since PTEN, a potent inhibitor of the PI3K/AKT pathway, and PPAR-α are direct targets of miR-21. Furthermore, the re-expression of PPAR-α and treatment with an agonist such as fenofibrate could increase PTEN and PDCD4 expressions and also induce FAO by increasing the expression of SLC22A5, CPT1, and ACOX1. In diabetic nephropathy, K-877, a novel PPAR-α agonist, accelerates FAO and inhibits FA synthesis by modulating the AMPK/ACC pathway [40]. 

**Figure 7:**
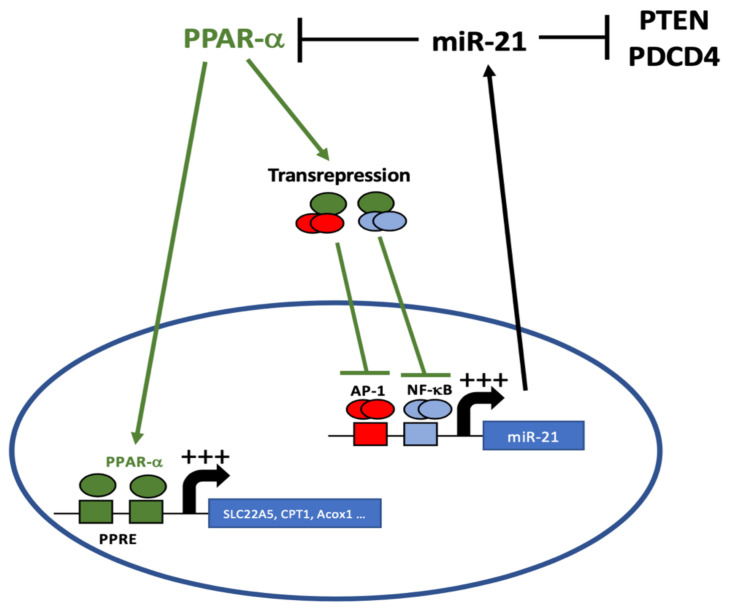
A double-negative feedback interaction between miR-21 and PPAR-α in clear renal cell carcinoma.

Previous studies have shown that transcriptional regulation mediated by transcription factors and the post-transcriptional control exerted by miRNAs are often highly coordinated [41,42,43]. In particular, the interplay between miRNAs and transcriptional regulators has been shown to regulate key developmental events or cell-fate decisions [41,42,43]. In particular, one well-described network motif, the double-negative feedback loop, is thought to regulate many binary cell-fate decisions [42,43,44]. This regulatory mechanism implies that one miRNA targets a transcriptional regulator, which in turn controls the expression of this miRNA and usually acts as a switch between two alternative cell states [42,43,44,45]. In this study, we propose that, in ccRCC, miR-21 and PPAR-α function in a double-negative feedback loop, where miR-21 and PPAR-α mutually repress each other to control lipid metabolism (Figure 7). In line with this, we showed that in vitro modulation of miR-21 likely induces metabolic changes by impacting PPAR-α expression and activity.

Many studies have linked miR-21 to poor prognosis and survival [29]. Previous studies have shown that the development of antisense oligonucleotides designed against miR-21 holds great promise for cancer therapy, and one anti-miR-21 drug candidate (RG-012) is already in the pipeline for clinical trials. In line with this, our data suggest that the proposed double-negative feedback interaction between PPAR-α and miR-21 could be therapeutically exploited to restore PPAR-α expression and consequently inhibit the oncogenic events mediated by the aberrant lipid metabolism of ccRCC. 

There were a few limitations in our study: (i) we did not perform in vivo experiments to support our in vitro results, and (ii) the measurement of FAO activity and lipid storage would further support the potential metabolic shift induced by miR-21 silencing and/or PPAR-α activation in ccRCC.

## 5. Conclusions

In this study, we propose that the lipid metabolic reprogramming of ccRCC occurs at least in part through a double-negative feedback loop between miR-21 and PPAR-α (Figure 7). miR-21 seems to be involved in the metabolic shift observed in renal cancer by targeting PPAR-α, which is one of the master regulators of lipid metabolism. In ccRCC, miR-21 silencing seems a promising strategy (i) to improve chemotherapy efficacy, (ii) to inhibit survival and anti-apoptotic pathways, and (iii) to reverse the metabolic shift toward FAO. This kind of strategy will open new therapeutic options to clinicians to ameliorate patient’s care.

## Data Availability

The data presented in this study are available on request from the corresponding author.

## Supplementary Materials

The following supporting information can be downloaded at: https://www.mdpi.com/article/10.3390/cancers14030795/s1, Figure S1: The original western blotting data.
