# Supplementary figures and images for: A Double-Negative Feedback Interaction between miR-21 and PPAR-α in Clear Renal Cell Carcinoma
Source: Cancers (Basel). 2022 Feb 4;14(3):795. doi: 10.3390/cancers14030795 (PMC8834244; doi:10.3390/cancers14030795)

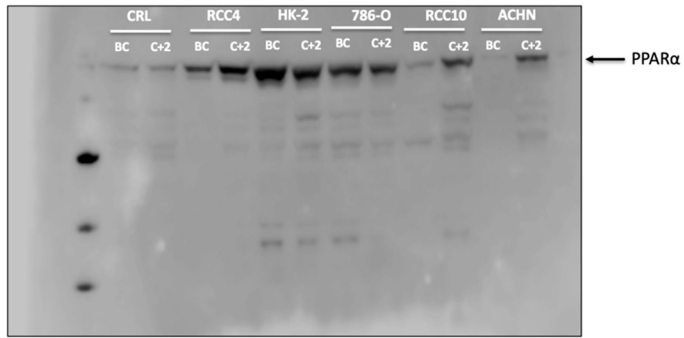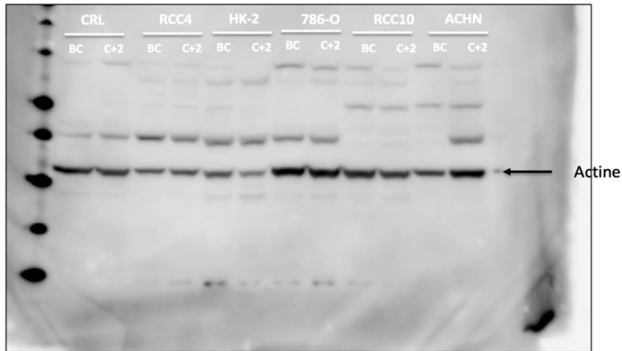

**ACHN**

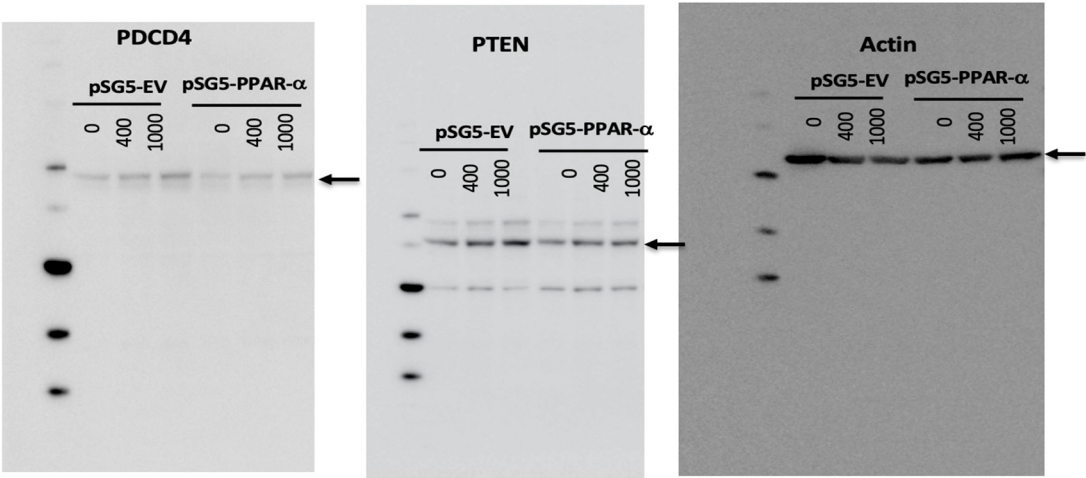

**786-O**

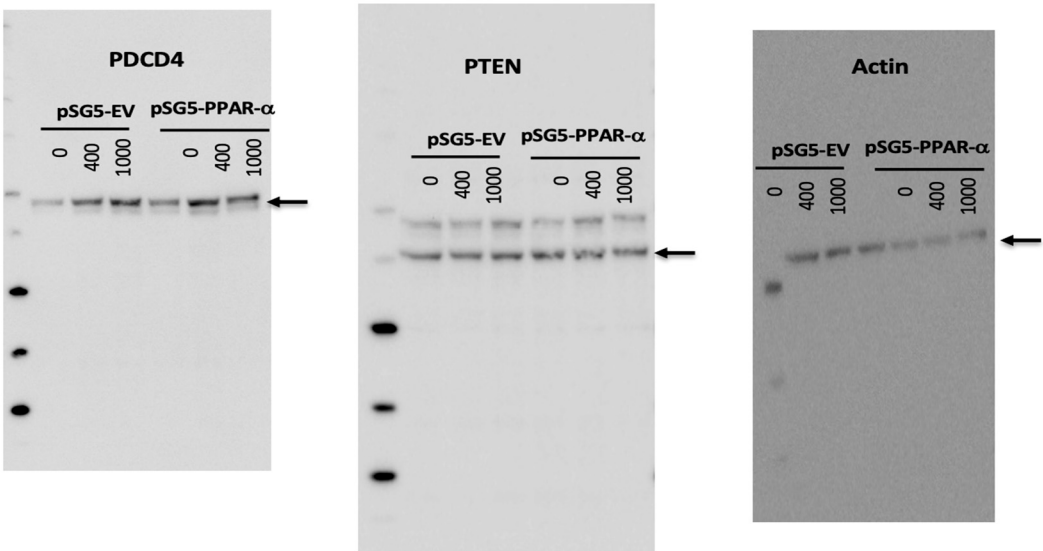

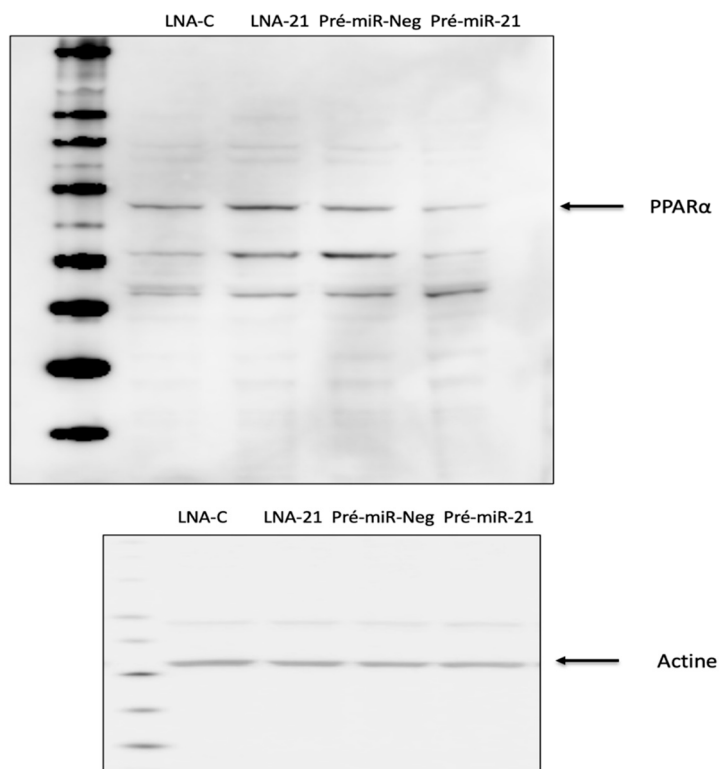

**Figure S1.** The original western blotting data.

Supplement: Supplementary file 1 [file cancers-14-00795-s001.zip › cancers-1544967-supplementary.pdf]
